# Naturally Equipped Urinary Exosomes Coated Poly (2−ethyl−2−oxazoline)−Poly (D, L−lactide) Nanocarriers for the Pre−Clinical Translation of Breast Cancer

**DOI:** 10.3390/bioengineering9080363

**Published:** 2022-08-03

**Authors:** Jiang Ni, Yuanyuan Mi, Bei Wang, Yuting Zhu, Yang Ding, Yongjuan Ding, Xia Li

**Affiliations:** 1 Department of Pharmacy, Affiliated Hospital of Jiangnan University, Wuxi 214000, China; jiangni16401@163.com (J.N.); miniao1984@163.com (Y.M.); xuewuhenwang@126.com (B.W.); tingting9189@126.com (Y.Z.); 2 College of Pharmacy, Pharmaceutical Series, China Pharmaceutical University, Nanjing 210000, China; dydszyzf@163.com

**Keywords:** breast cancer, membrane−coated biomimetic nanoparticles, doxorubicin, urinary exosomes, poly (2−ethyl−2−oxazoline)−poly (D, L−lactide)

## Abstract

Recently, biomimetic nanoparticles for tumor−targeted therapy have attracted intensifying interest. Although exosomes are an excellent biomimetic material, numerous challenges are still hindering its clinical applications, such as low yield, insufficient targeting efficiency, and high cost. In this work, urinary exosomes (UEs) with high expression of CD9 and CD47 were extracted from breast cancer patients by a non−invasive method. Here, a nanotechnology approach is reported for tumor homologous targeting via CD9 and phagocytosis escape via CD47 through UE−coated poly (2−ethyl−2−oxazoline)−poly (D, L−lactide) (PEOz−PLA) nanoparticles (UEPP NPs). The cytotoxic agent doxorubicin (DOX)−loaded UEPP (UEPP−D) NPs with an initial particle size of 61.5 nm showed a burst release under acidic condition mimicking the tumor microenvironment. In vitro experiments revealed that UEPP−D NPs exhibited significantly improved cellular uptake, cytotoxicity, and apoptosis in MCF−7 cell lines as compared to DOX−loaded PEOz−PLA nanoparticles (PP−D NPs) and free DOX. More importantly, anti−phagocytosis and pharmacokinetic studies confirmed that UEPP−D NPs had superior immune escape ability and significantly prolonged the drug’s bloodstream circulation in vivo. Finally, UEPP−D NPs showed a markedly higher antitumor efficacy and lower side−toxicity in MCF−7 tumor bearing nude mice model. Thus, this versatile nano−system with immune escape, homologous targeting, and rapid response release characteristics could be a promising tool for breast cancer treatment.

## 1. Introduction

Given the huge systemic sensitivities of many chemotherapeutic agents, traditional chemotherapy for breast cancer treatment is only moderately effective at treating and extending the patients’ lives [1,2,3,4]. Moreover, low amounts of anticancer agents can reach the target sites which are insufficient for effective apoptosis [5]. Nanoparticles displayed promising therapeutic effect against cancer owing to the enhanced permeability and retention (EPR) effect, laying the foundation for passive targeting [6,7,8,9,10,11]. To ensure efficient accumulation and tumor targeting, nanoparticles are further functionalized with active targeting moieties, such as peptides, antibodies, or other biomolecules [8,9,10,11,12]. However, these latter can cause immune response upon administration into the body [13,14,15,16,17]. Furthermore, the expense of these specific ligands is generally quite high, posing a problem for large−scale manufacturing. Therefore, the creation of reliable, biodegradable, and cost−effective nano−systems is still an unmet need of the current breast cancer chemotherapy.

Recently, exosome−based biomimetic nanoparticles have acquired considerably enormous attention as efficient drug delivery systems. Exosomes are extracellular vesicles secreted by nearly all cells with an average size ranging from 40 to 200 nm [18,19,20,21] and have been used as attractive biomimetic materials owing to their biocompatibility, longevity in circulation, low immunogenicity, and side−toxicity. Furthermore, exosomes have shown higher cellular uptake and targeting abilities owing to the proteins expressed on their surfaces. For example, tetraspanin protein CD9 and CD81 trigger tumor−specific delivery by the fusion of the exosomes cell membranes to the target cell membranes, integrin−associated glycoprotein CD47 and CD63 help in escaping the phagocytosis through the “Don’t eat me” signal, making exosomes getting rid of macrophages recognition, phagocytosis, and augment homogenous endocytosis, while CD55 and CD59 inhibit complement attack [1,19,22]. However, exosomes are usually isolated from in vitro cell breeding medium, such as tumor or immune cell breeding medium. In a number of studies, the yield was very low and insufficient for therapeutic use [23]. Moreover, non−specific tumor targeting and contamination in extraction process are also major drawbacks of these exosomes that limit their clinical use [23,24,25]. Consequently, developing an efficient approach to construct exosome−based biomimetic nanoparticles with low cost, high purity, and specific tumor targeting ability becomes desirable for potential clinical translation.

Urine collected from breast cancer patients provide a non−invasive and easily available resource of urinary exosomes (UEs) that can meet the large−scale need for therapeutic use [1,19,23,25]. Using UEs might be helpful to minimize harmful cross−contamination during the long−term culture of exosomes−budding cells. Interestingly, exosomes obtained from the patients’ own urines served as endogenous delivery systems that are expected to display improved safety and effectiveness, and less immunogenicity. Moreover, UEs from breast cancer patients present similar membrane antigens to cancer cell, such as E−cadherin and CD 47, thereby providing “Trojan horses” for specific drug delivery to tumor site. Most importantly, UEs from breast cancer patients carry more abundant key proteins (CD63, CD9, and CD47) as compared to that from normal people, which are essential for long blood circulation, immune escape and tumor targeting [1,23,26]. In view of the above, we believe that UE−based biomimetic nanoparticles could provide a new nanoplatform for individualized therapy.

The hydrophilic polymer “poly(2−ethyl−2−oxazoline) (PEOz)” was previously found to be very well ionized at acidic pH of tumor microenvironment. Moreover, PEOz offers remarkable physiological biocompatibility, making it an auspicious substitute for PEG [27]. The poly(2−ethyl−2−oxazoline)−poly(D,L−lactide) (PEOz−PLA) was also previously synthesized and successfully employed for drug delivery [28]. A group of studies have reported the efficient use of PEOz as stimuli−sensitive drug carrier and revealed the ionic behavior of PEOz in endosomal/lysosome’ pH conditions [29,30,31]. These characteristics make PEOz−PLA a promising encapsulating biodegradable and biocompatible material to deliver cytotoxic drug to tumor cells.

**Scheme 1:**
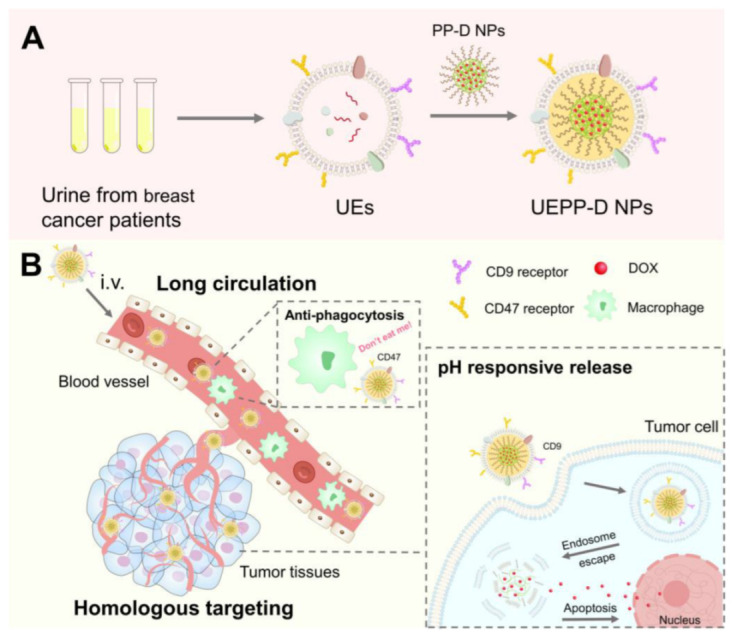
Representative illustration of UEPP−D NPs for the treatment of breast cancer. (**A**) Preparation of UEPP−D NPs. (**B**) Schematic of enhanced DOX delivery by the UEPP−D NPs for cancer therapy. UEPP−D NPs exert long circulation time in the blood stream and escape phagocytosis via upregulated UE−CD47 through the “Don’t eat me” mechanism. Subsequently, UEPP−D NPs efficiently target tumor cells via upregulated UE−CD9. The pH−sensitive release from PEOz−PLA results in adequate accumulation of DOX in the tumor cells and nucleus, causing heightened apoptosis.

Herein, we emphasized that UE−coated PEOz−PLA nanoparticles can serve as homologous tumor−specific targeting carrier for cytotoxic agents such as doxorubicin (DOX), where pH−sensitive drug release can enable tumor specific release of the drug [5,28]. DOX was encapsulated in PEOz−PLA nanoparticles and then coated with UEs to fabricate UEPP−D NPs. The exosome−biomimetic UEPP−D NPs possessed prolonged blood circulation, prominent tumor accumulation efficiency, and markedly improved the tumor inhibitory effect of DOX in vivo (Scheme 1).

## 2. Experimental

### 2.1. Materials

Doxorubicin hydrochloride (DOX·HCl) was purchased from Beijing Huafeng United Technology Co. (Beijing, China). PLA5000−b−PEOz2000 (PEOz−PLA, Cat. R−PL7001) was obtained from Xi’an ruixi Biological Technology co.,Ltd (Xi’an, China). 1,1′−dioctadecyl−3,3,3′,3′−tetramethylindotricar−bocyanine iodide (DiR), IR780 and Hoechst 33,342 were all provided by Thermo Fisher Scientific, Waltham, MA, USA. DMEM medium was purchased from Gibco (Tulsa, OK, USA). CD81 antibody (Cat. 66866−1−Ig), CD47 antibody (Cat. 66304−1−Ig), and CD9 antibody (Cat. 20597−1−AP) were all provided by Proteintech (Wuhan, China). All other reagents were obtained from Sigma−Aldrich (St. Louis, MO, USA) unless otherwise stated.

### 2.2. Preparation of UEs

Urine samples were collected from breast cancer patients and debris were removed by centrifugation at 3000× *g* for 30 min at 4 °C. First, UEs were extracted and purified by centrifuging two times at 150,000× *g* for 60 min under 4 °C. UE pellets were collected and re−suspended in PBS [19]. Then, the solution was filtered through 0.22 μm pore size membrane (Millex−GV, Millipore, Burlington, MA, USA) to eliminate residual cell debris or bacteria. Thereafter, the purified exosomes were re−suspended in sterilized PBS and stored at −80 °C for further use. The protein contents of the isolated pure exosomes were calculated by BCA protein assay [32].

### 2.3. Preparation of UEPP−D NPs

Amphiphilic PEOz−PLA (PP) di−block copolymer was successfully employed for the synthesis of pH−sensitive NPs. The PP−D NPs were prepared using a thin−film hydration method. First, 4 mg DOX (pre−dissolved in 10% DMSO) and 20 mg PEOz−PLA were dissolved in methanol (20 mL), the mixture was then evaporated under vacuum at 60 °C. Thereafter, the resulting stripped film was hydrated using 5 mL of deionized water at 60 °C and the mixture was further vortexed for 5 min. The unloaded DOX was removed by filtering the NPs suspension through a 0.22 μm pore size membrane [25,26,28]. For synthesis of DiR−loaded NPs (PP−DiR NPs), 20 mg PEOz−PLA and 1 mg DiR (pre−dissolved in 10% DMSO) were dissolved in methanol (20 mL). For synthesis of IR780−loaded NPs (PP−IR780 NPs), 20 mg PEOz−PLA and 1 mg IR780 (pre−dissolved in 10% DMSO) were dissolved in 20 mL of methanol, then treated as mentioned above [33].

For coating UEs, 1 mg pure UEs dissolved in PBS was mixed with 10 mg PP NPs under vortex at 4 °C, then allowed to react the whole night at 4 °C and afterwards the mixture was extruded through 200 nm and 100 nm polycarbonate membranes by a mini extruder (Avanti Polar Lipids Inc. Alabaster, AL, USA). The NPs were collected through centrifugation (30,000× rpm, 20 min) and lyophilized for storage and characterization.

### 2.4. Western Blot Analysis

Western blot (WB) assay was carried out to measure the protein biomarkers of the UEs. First, the lysates of UEs were obtained using RIPA buffer, then analyzed using 10% SDS−PAGE. The proteins were transferred onto PVDF membranes using the Bio−Rad Trans−Blot^®^ Cell (Hercules, CA, USA) for 1 h at 4 °C, then blocked using 5% non−fat dehydrated milk for 2 h at 37 °C. Finally, the obtained blots were then stained with anti−CD9, anti−CD81, and anti−CD47 antibodies [1].

### 2.5. Characterization

The size, appearance, and morphology of UEs and UEPP−D NPs were evaluated using transmission electron microscopy (TEM) (JEM−2010, JEOL, Tokyo, Japan). The hydrodynamic size of PP−D NPs and UEPP−D NPs were determined by dynamic light scattering (DLS) method (Malvern zeta sizer). The PDI and zeta−potential were also measured by DLS. The drug loading of DOX was measured using fluorescence microplate reader.

### 2.6. Drug Release

A total of 2 mL of UEPP−D NPs solution (300 μg DOX content) was transferred to a dialysis bag (MWCO = 3500) and then dipped into 50 mL conical flask containing 20 mL of 50 mM PBS at different pH values (5.0, 6.5, and 7.4) with 1% SDS. The flasks were kept constantly shaking (150 rpm) in a shaking water bath at 37 °C. At specific time points, 1 milliliter of the release medium was collected and replaced with an equal volume of fresh buffer. The concentration of released DOX was determined using a fluorescence microplate reader.

### 2.7. Cell Culture and Cellular Uptake Assay

The cellular uptake of UEPP−D NPs in human breast cancer MCF−7 cell line was measured using fluorescence microscopy (Ti−S, Nikon, Tokyo, Japan) and flow cytometry (FACS Verse, BD, USA). MCF−7 cells were grown in DMEM culture medium containing 10% FBS and penicillin/streptomycin (1%) at 37 °C in 5% CO_2_. Then, the cells were seeded in 24−well plates (5 × 10^4^ cells/well). After 24 h, the cells were washed twice with PBS, then treated with DOX, PP−D NPs, and UEPP−D NPs (DOX3 µg/mL) for 1, 2, and 4 h, respectively. Subsequently, the cells were carefully washed thrice with cold PBS containing 4% paraformaldehyde (PFA) for 20 min, then washed again with cold PBS. Afterwards, the cells were further incubated with Hoechst 33,342 (10 µg/mL) for 10 min, and then imaged using fluorescence microscopy.

For flow cytometry analysis, MCF−7 cell lines were grown in 6−well plates (4 × 10^4^ cells/well) for 24 h. After the morphology became normal, cells were washed twice with PBS (10 mM, pH 7.4). Subsequently, 2 mL of DOX, PP−D NPs, and UEPP−D NPs (DOX 3 µg/mL) were added followed by 1, 2, and 4 h incubation. The cells were washed thrice with cold PBS then digested with trypsin (0.5 mL/well for 90 s). Thereafter, the cells were pelleted by centrifugation at 1500 rpm for 5 min, washed two times with PBS, then re−suspended in 0.5 mL of PBS for FACS analysis.

### 2.8. Cell Viability Assay

MCF−7 cells were grown in 96−well plates (3500 cells/well) and incubated for 48 h. The medium was then removed and the cells were washed thrice with PBS. In the case of biomaterial MTT assay, cells were incubated with blank UEPP NPs with the concentration ranging from 0.005 to 20 mg/mL for 48 h. To perform MTT assay for DOX−loaded nanoparticles, various concentrations of free DOX, PP−D, and UEPP−D NPs were added followed by 48 h incubation. About 20 µL MTT solution (5 mg/mL) was added to each well then incubated for 4 h. The medium was then aspirated, and 150 µL of DMSO was added to each well followed by gentle shaking for 10 min. Thereafter, the absorbance (OD) was detected at 490 nm and cell viability was calculated.

### 2.9. Apoptosis Study

The apoptosis rate was quantified by flow cytometry analysis, as previously described. MCF−7 cells were grown in 6−well plates (1 × 10^5^ cells/well) for 24 h. After the morphology became normal, cells were washed twice with PBS (10 mM, pH 7.4). Subsequently, 2 mL of DOX, PP−D NPs, and UEPP−D NPs (DOX 3 µg/mL) were added followed by 1, 2, and 4 h incubation. The cells were washed thrice with cold PBS then digested with trypsin (0.5 mL/well for 90 s). Thereafter, the cells were pelleted by centrifugation at 1500 rpm for 5 min, washed two times with PBS, then re−suspended in PBS (0.5 mL) for flow cytometry quantification of apoptosis.

### 2.10. In Vivo Tumor Targeting

All in vivo experiments were carried out in accordance with the guidelines of medical ethics committee of Jiangnan University, Wuxi, China. Female Balb/c nude mice (weighing 18–22 g) acquired from Laboratory Animal Centre of Jiangnan University (Wuxi, China), and kept under standard housing conditions. To develop subcutaneous breast cancer murine model, 3 × 10^6^ MCF−7 cells in 100 μL of PBS were injected subcutaneously into the mouse proximal femur region [34].

The bio−distribution and imaging studies were performed using IVIS lumina system. MCF−7 tumor bearing mice were randomly divided to three groups that were intravenously injected with DiR, PP−DiR NPs, and UEPP−DiR NPs (0.4 mg DiR/kg, *n* = 4), respectively. Thereafter, at predetermined time points (post−injection, 6, 12, and 24 h) the mice were anesthetized with isoflurane then imaged using IVIS Lumina LT Series III (PerkinElmer, Waltham, MA, USA). At 24 h post−injection, the mice were euthanized and the major organs and tumors were harvested. Afterwards, the region−of−interest (ROI) analysis was performed to determine the fluorescence intensity using Living Image 3.0 software (PerkinElmer).

### 2.11. Anti−Phagocytosis and Pharmacokinetics Study

To study the phagocytosis escape phenomenon of UEPP NPs, RAW 264.7 macrophages were treated with PP−D NPs and UEPP−D NPs for 4 h at a DOX concentration of 2 μg/mL. The fluorescence intensity of RAW264.7 cells was quantitatively analyzed via flow cytometry [32]. The experiment was performed in triplicate.

For quantitative determination of pharmacokinetics study, normal ICR mice (weighing 20–25 g) were assigned and divided into three groups that were intravenously treated with free IR780, PP−IR780 NPs, and UEPP−IR780 NPs (1 mg IR780/kg, *n* = 5), respectively. Blood samples (20 μL) from the tail of nude mice were collected at 1 min, 2, 4, 8, 12, 24, 48, and 72 h after injection. The samples were then mixed with 180 μL DMSO to extract IR780 and transferred to a 96−well plate to quantify the NIR fluorescence intensity using Living Image 3.0 at 780/817 nm.

### 2.12. In Vivo Therapeutic Efficacy and Side Toxicity

Tumor model was developed in female Balb/c nude mice as described earlier. As the tumor size reached roughly 100 mm^3^, the tumor bearing nude mice were randomly divided into four groups that were injected intravenously with saline, free DOX, PP−D NPs, and UEPP−D NPs (5 mg DOX/kg, *n* = 5) every other day for two weeks. The mice behavioral change, tumor size, and body weight were noted every other day. The tumor size was calculated using the equation: Tumor size = L × (W)^2^/2, where L and W represent the length and width of tumors, respectively. After 15 days, serum samples were collected for the determination of the major organs function enzymatic markers, i.e., liver (AST, ALT), heart (CK), and kidney (BUN, CRE). The mice were then sacrificed, and major organs and tumors were harvested then fixed in 10% formalin for histopathological examination.

### 2.13. Statistical Analysis

The results are displayed as mean ± SD. All the results were compared by two tailed student’s *t* test (unpaired or paired). Where ANOVA was applied, when there was multiple comparison of more than two groups. The *p*-value lower than 0.05 was considered statistically significant.

## 3. Results and Discussion

### 3.1. Preparation and Characterization of UEPP−D NPs

To achieve tumor−responsive release and simpler fabrication process, membrane−encapsulated nanoparticles rather than drug−loaded exosomes were selected. In this study, UEs from breast patients were used as the biomimetic membrane material for coating PP NPs. We successfully extracted high−purity UEs according to the previously reported method [1,23]. Subsequently, PP−D NPs were prepared using the classic volatilization thin−film hydration method. Finally, UEs were coated onto PP−D NPs through mechanical extrusion to obtain the UEPP−D NPs. Surprisingly, approximately 1.7 mg of UEs was collected from 200 mL of urine. In comparison, only 50 μg of cell−derived exosomes could be extracted from 200 mL of DMEM in our preliminary data. Thus, the yield of UEs can be satisfied for the large−scale production of nanoparticles [26].

**Figure 1:**
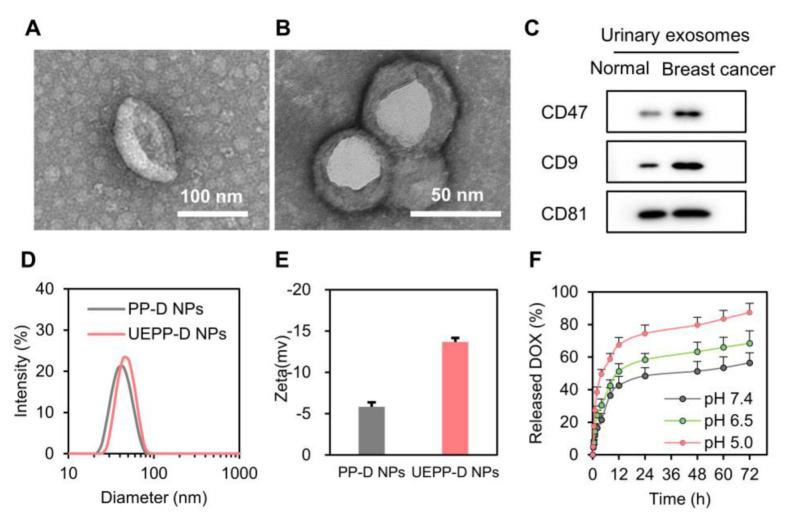
Characterization of UE and UEPP−D NPs. (**A**) TEM images of UE. (**B**) TEM images of UEPP−D NPs. (**C**) Western blot analysis of UE proteins. (**D**) Hydrodynamic size of the NPs (*n* = 3). (**E**) Zeta−potential of NPs (*n* = 3). (**F**) DOX release from UEPP−D NPs at various pH (*n* = 3). Data are presented as mean ± SD.

**Figure 2:**
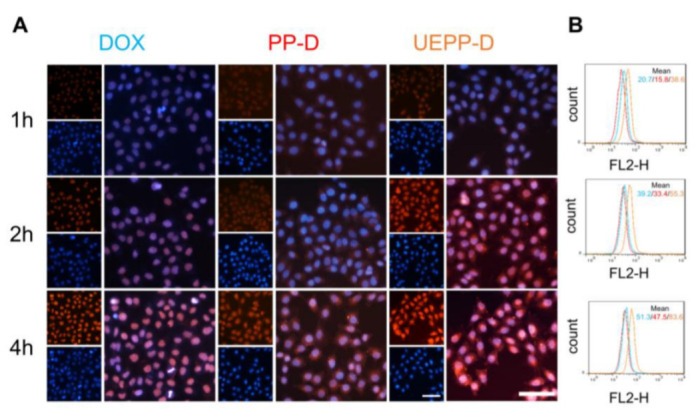
Cellular uptake investigation. (**A**) Fluorescence microscopy images of free DOX, PP−D NPs, and UEPP−D NPs incubated in MCF−7 cells for 1 h, 2 h, and 4 h, Scale bar is 100 μm. (**B**) Flow cytometry quantification. Blue, red, orange color histograms signify the MCF−7 cells incubated with free DOX, PP−D NPs, and UEPP−D NPs in MCF−7 cells, respectively. (*n* = 3). Data are presented as mean ± SD.

The loading efficiency of DOX in PP−D NPs and UEPP−D NPs were 9.46% and 8.73%, respectively. The size and appearance of UEs and UEPP−D NPs were evaluated under TEM [35,36]. UEs showed a round−like shape with an average size of 72.4 nm (Figure 1A). Moreover, UEPP−D NPs displayed core−cell structures with a diameter of around 47.2 nm (Figure 2A), while the outer exosomal membrane thickness was about 11.4 nm, as reported previously [32,37]. The hydrodynamic diameter of PP−D NPs and UEPP−D NPs was determined using DLS (Figure 1D,E). The average size of UEPP−D NPs was about 61.5 nm, slightly larger than that of PP−D NPs (47.5 nm). The PDI values of PP−D NPs and UEPP−D NPs were less than 0.2 showing uniform size distribution of the nanoparticles (Figure S1 in Supplementary Materials). Zeta−potential of UEPP NPs was found to be negative, which might be due to negatively charged exosomal membrane.

Membrane proteins of urinary exosomes from healthy controls and from breast cancer patients were identified by Western blot assay. Pre−experiments confirmed that there was a linear relationship between the protein amounts of loaded exosomes and the intensity of the protein bands. The results showed that CD81 is highly expressed both in normal people and breast cancer patients with similar expression levels (Figure 1C). Interestingly, the expression levels of CD9 and CD47 in breast cancer patients were obviously higher than those in normal people (Figure 1C). These results are in accordance with previous reports [20] and reveal that UEPP−D NPs can efficiently target the tumor cells by virtue of CD9 while CD47 up−regulation exposed the fact that UEPP−D NPs can be able to escape the macrophages uptake [1]. The up−regulation of these membrane proteins in UEs might be the key factor for homologous tumor targeting and anti−phagocytosis.

The release profile of DOX from UEPP−D NPs was determined in different pH conditions (0.01 M PBS at pH 5.0, pH 6.5, and pH 7.4). As shown in Figure 1F, the release was found to be maximum at pH 5.0, showing 74.45% of the DOX release at 24 h, where the release was 58.39% and 48.45% at pH 6.5 and 7.4, respectively. These results indicate that the pH−responsiveness of UEPP NPs display selective drug release characteristics upon reaching the mildly acidic condition in the tumor microenvironment (pH 6.5) and endo−lysosomal structures after internalization (pH 5.0). Overall, these results indicate the successful preparation of UEPP−D NPs.

### 3.2. In Vitro Cellular Uptake and Homologous Targeting Analysis

The in vitro targeting ability and uptake efficacy of UEPP−D NPs were evaluated using fluorescence microscopy and flow cytometry. MCF−7 cells were treated with free DOX, PP−D NPs, and UEPP−D NPs for 1, 2, and 4 h, respectively. Thereafter, the cells nuclei were stained with hoechst33352, a blue fluorescence dye. As displayed in Figure 2A, the red fluorescence (indicating DOX) in free DOX group appeared in the nuclei and increased with time. In comparison, the red fluorescence signal in PP−D NPs and UEPP−D NPs groups was also noticed in the cell nuclei from 2 h and gradually improved with increasing the incubation time, indicating the rapid release of DOX from PP−D NPs and UEPP−D NPs, thereby entering the nuclei as free DOX. The intracellular signal of DOX in free DOX group was somewhat stronger than PP−D NPs group, which is likely related to the easy diffusion of free DOX into the cancer cells. It is noteworthy that the mean fluorescence intensity (MFI) of DOX in UEPP−D NPs group was about 1.63- and 1.76-fold higher than that in free DOX and PP−D NPs groups at 4 h, respectively (Figure 2B). These results indicate that the intracellular delivery of DOX was enhanced using UE membranes for nanoparticles coating. The efficient cell endocytosis of DOX via UEPP−D NPs can be attributed to their biocompatibility and specific proteins expressed on the surface (such as CD9 and CD81) of UEs. According to previous reports, exosomes can transport through the cell membrane to enter the cells via fusion and/or membrane proteins−mediated internalization, thereby delivering the loaded drugs into the targeted cells [36]. These results suggest that UEPP−D could enhance the tumor accumulation of DOX by efficient internalization via UE−proteins and rapid DOX release via PEOz.

### 3.3. In Vitro Anti−Tumor Analysis

**Figure 3:**
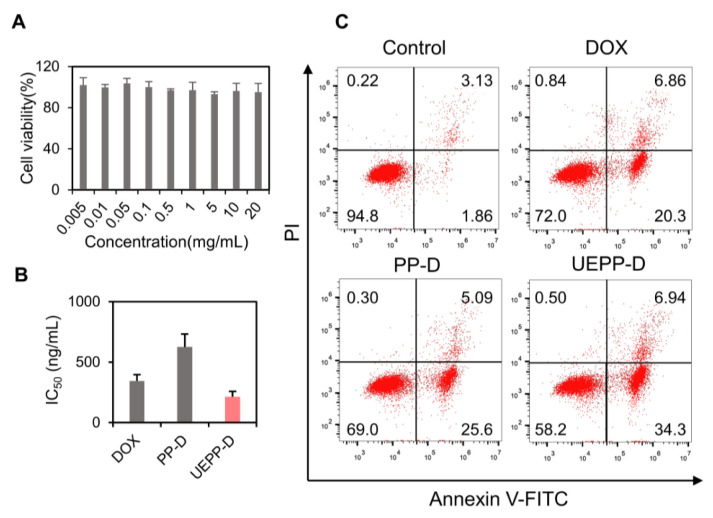
Evaluation of the in vitro cytotoxic and apoptotic effects of UEPP−D. (**A**) MTT assay of blank UEPP NPs in MCF−7 cells, where the NPs were incubated for 48 h in MCF−7 cells (*n* = 6), (**B**) IC_50_ value for free DOX, PP−D NPs, and UEPP−D NPs calculated by MTT assay (*n* = 3), and (**C**) rate of apoptosis quantified by flow cytometry for control, free DOX, PP−D NPs, and UEPP−D NPs (*n* = 3). Data are presented as mean ± SD.

In vitro anti−tumor analyses were conducted by performing MTT assay and flow cytometric apoptosis assay. In Figure 3A, blank UEPP NPs showed no obvious toxicity. The IC_50_ was measured for free DOX, PP−D NPs, and UEPP−D NPs after 48 h incubation. The value was 345 ng/mL, 625 ng/mL, and 213 ng/mL for free DOX, PP−D NPs, and UEPP−D NPs, as reported in Figure 3B. The rate of apoptosis was calculated by flow cytometry, where ~27% apoptotic cells were found for free DOX. While almost 41% and 32% apoptosis were found, when cells were treated with UEPP−D and PP−D NPs, respectively (As shown in Figure 3C). The increased rate of apoptosis might be due to the fact that UEPP−D NPs can remarkably target the MCF−7 cells via CD9 and rapidly release in acidic endosomes, resulting enhanced accumulation and cytotoxicity in tumor cells. Therefore, these data confirm that the in vitro anti−tumor effect of DOX was enhanced with UEPP−D NPs.

### 3.4. In Vitro Anti−Phagocytosis, In Vivo Tumor Targeting, and Pharmacokinetics Study

**Figure 4:**
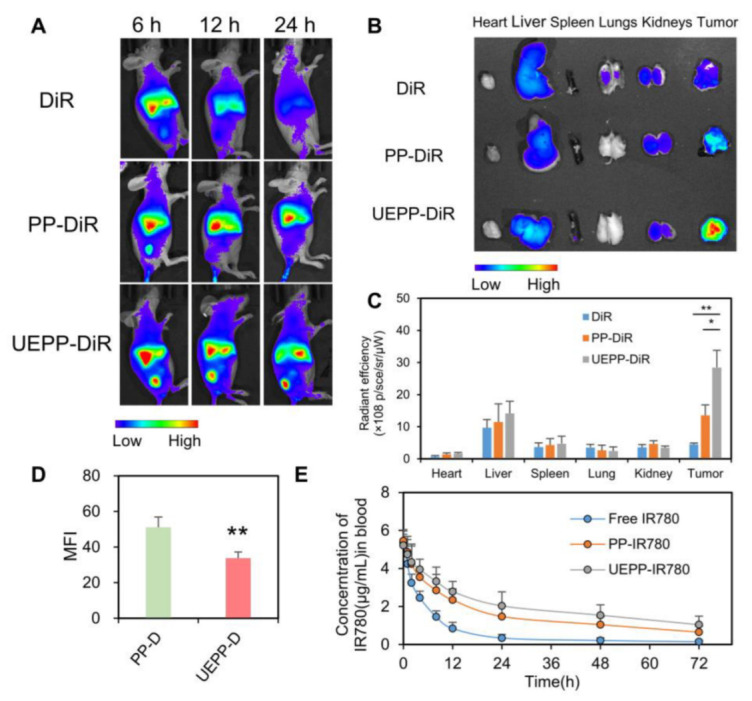
Biodistribution in tumor−bearing nude mice and pharmacokinetics. (**A**) Ex vivo IVIS image of tumor−bearing nude mice of various groups, (**B**,**C**) in vivo IVIS images of harvested major organs and tumors. The radiant efficiency was calculated by IVIS Lumina 3.0 (*n* = 3), and (**D**) evaluation of macrophages engulfment of PP−D NPs, and UEPP−D NPs, and (**E**) plasma concentration curve (*n* = 4). Data are presented as mean ± SD. Where *p* < 0.05 (*), *p* < 0.01 (**).

To explore whether the UEPP NPs had anti−phagocytosis ability, the cellular uptake of PP−D NPs and UEPP−D NPs in RAW 264.7 macrophages was determined using flow cytometry. As displayed in Figure 4D, the MFI of DOX in UEPP−D NPs decreased significantly (*p* < 0.01) compared to that in PP−D NPs. These results are likely related to the high expression of CD47 in UEs that could be recognized by signal regulatory proteins, thereby releasing the “Don’t eat me” signal and blocking phagocytosis [23].

The targeting capability and biodistribution profile of free DiR, PP−DiR NPs, and UEPP−DiR NPs were estimated in the MCF−7 murine breast cancer model (Figure 4A). The free DiR showed lower fluorescence signals at the tumor site, which is likely related to the rapid renal clearance. In contrast, UEPP−DiR NPs displayed highest tumor accumulation ability, wherein significantly stronger signals were noticed within the tumor at 6 h post−injection and the highest levels were achieved at 24 h post−injection. The major organs and tumor tissues were then harvested at 24 h post−injection to detect the fluorescence signals of DiR (Figure 4B,C). The mean fluorescent intensity of harvested tumors revealed prominent accumulation of UEPP−DiR NPs compared with free DiR and PP−DiR NPs, wherein UEPP−DiR NPs group displayed 2.1-fold stronger signal as compared to PP−DiR NPs group. These results indicate the higher tumor accumulation and targeting abilities of UEPP−DiR NPs in vivo. While there was uniform distribution of PP−DiR NPs and UEPP−DiR NPs in all other major organs that probably revealed the equivalent dose administration of DiR [15]. Therefore, the in vivo distribution study reveals that UEs from breast cancer patient can be efficiently employed for selective targeting of breast cancer cells and can be a useful approach for potential clinical application.

The pharmacokinetic study was carried out to evaluate the blood circulation time of PP−IR780 NPs and UEPP−IR780 NPs with respect to free IR780. The blood plasma profile showed extended blood circulation time for PP−IR780 NPs and UEPP− IR780 NPs, as compared to free IR780. Next, we determined the pharmacokinetic parameters given in Table 1. As shown in Figure 4E and Table 1, both PP NPs and UEPP NPs achieved relatively higher concentration levels of IR780 that were detectable up to 48 h post−injection. In contrast, lower concentration levels were noticed with free IR780 group then became undetectable after 24 h post−injection. The extended circulation time of NPs might be accredited to the long circulation capability of PEOz comparable to PEG chains. Moreover, compared with those of the PP−IR780 group, the area under the plasma concentration curve (AUC), mean residence time (MRT), and plasma half−life (t_1/2_) of UEPP−IR780 NPs group were 1.39-, 1.17-, and 1.13-fold higher, respectively.

### 3.5. In Vivo Therapeutic Efficacy and Systemic Toxicity

**Figure 5:**
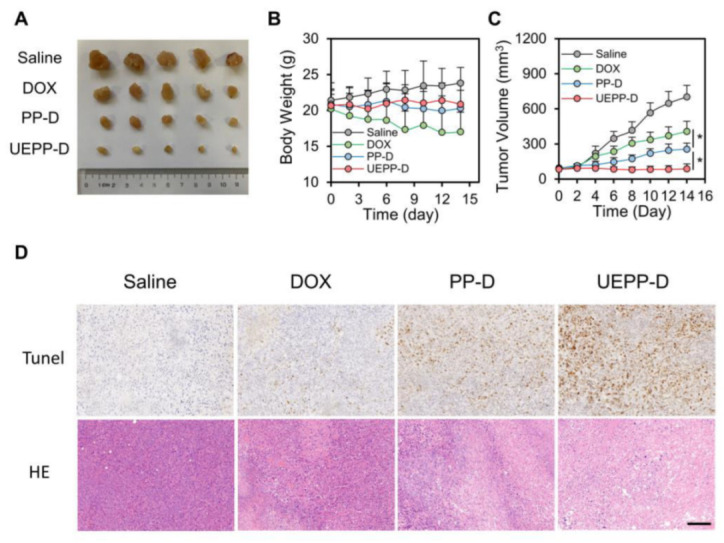
In vivo therapeutic efficacy. (**A**) Images of the excised tumors after treatment with various groups. (**B**,**C**) Body weights and relative tumor volume of excised tumors treated with various groups (*n* = 5). (**D**) H&E and TUNEL staining of excised tumor sections treated with various groups PP−D NPs, and UEPP−D NPs. Scale bar is 100 μm. Data are presented as mean ± SD. Where *p* < 0.05 (*).

To further support their advantageous efficiency, the in vivo antitumor efficacy of UEPP−D NPs was evaluated using MCF−7 orthotropic tumor murine model. For this purpose, tumor−bearing mice were randomly assigned into four groups then treated intravenously with saline, free DOX, PP−D NPs, and UEPP−D NPs. The tumor volume was measured every other day. The results showed that UEPP−D NPs markedly inhibited the tumor growth as compared to PP−D NPs and free DOX. On day 15, tumor tissues from different groups were harvested, weighed, and photographed, then their relative tumor volume was measured (Figure 5A). Of note, the highest inhibition rate (87.6%) was observed with UEPP−D NPs as compared to DOX and PP−D NPs (IR = 42.1% and 63.6%, respectively). That echoes the tumor targeting aptitude of UEPP−D NPs, and augmented accumulation of UEPP−D NPs integrated to the significant inhibition of the tumor growth (Figure 5C). No significant variations in the body weight changes were noticed in the UEPP−D group, revealing an apparent systemic safety [38]. The in vivo treatments revealed the effective anti−tumor activity of the UEPP−D NPs. Besides the strong anti−tumor efficiency, the H&E and TUNEL staining were performed to estimate the tumor cytotoxicity and ratio of apoptotic cells. As shown in Figure 5D, the light regions in the tumor sections revealed amplified toxic effects to the tumor cells by UEPP−D NPs as compared with control saline, as well as free DOX and PP−D NPs. Figure 5D also showed the increased number of apoptotic cells for UEPP−D NPs as compared to saline, free DOX, and PP−D NPs ensuring the improved therapeutic effects of UEPP−D NPs.

The above−stated results provide strong evidence of the superior antitumor efficacy of UEPP−D NPs. This could be attributed to their higher tumor accumulation owing to the active targeting ability of UEPP−D NPs toward the tumor cells (via proteins−mediated homologous targeting and rapid internalization). Furthermore, due to the highly expressed CD47 receptor on the surface of UE−coated nanoparticles, UEPP−D NPs exhibit superior long blood circulation and immune escape effect. Moreover, the surface charge inversion upon achieving the tumor microenvironment/tumor endosome by PEOz chains in UEPP−D NPs could lead to the rapid release of DOX, thereby enhancing cell apoptosis.

**Figure 6:**
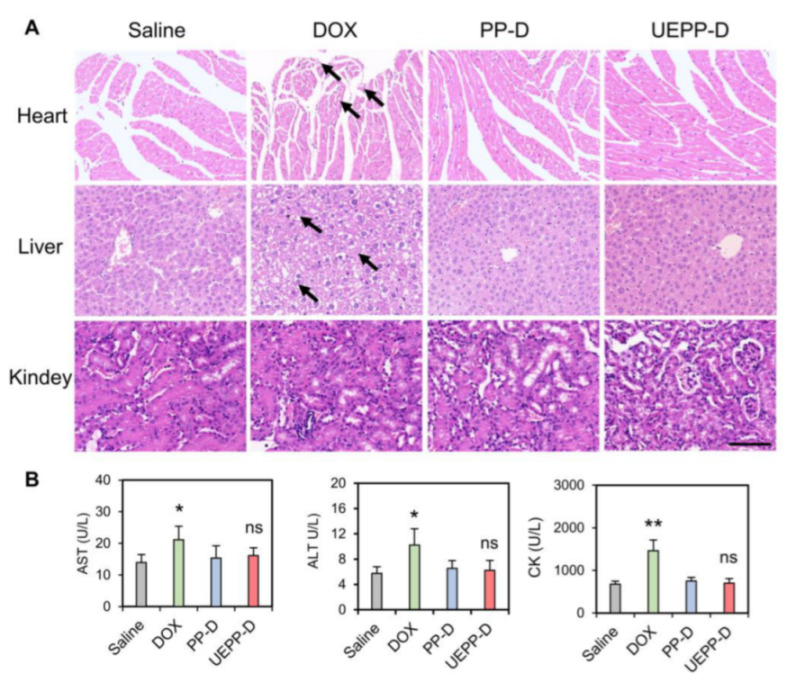
Evaluation of systemic toxicity. (**A**) H&E staining of the major organs from different groups. Black arrows indicate organ damages. (**B**) Serum levels of AST, ALT, and CK treated with various groups (*n* = 5). Scale bar is 100 μm. Data are presented as mean ± SD. Where *p* < 0.05 (*), *p* < 0.01 (**), ns means not significant, saline versus other groups.

The preliminary estimation of the drugs side toxicity was evaluated through monitoring the changes in body weight, mice behavior, and histopathological examination of major organs (liver, heart, and kidney) [17]. In addition, serum concentrations of enzymatic markers of the liver (AST, ALT), heart (CK), and kidney (BUN and CRE) functions were detected in different treatment groups [35]. As shown in Figure 5B, mice treated with free DOX showed noticeable weight loss and abnormal activity comparing to other groups, which is likely due to the free drug’s systemic toxicity. In addition, increased serum levels of AST, ALT, and CK were observed with this treatment (Figure 6B), while H&E staining revealed some liver inflammatory alteration (infiltration of inflammatory cells and mild hepatocytes necrosis) and dissolved cardiac muscle fibers (Figure 6A). These results could be explained by the non−specific distribution of free DOX in different organs. Interestingly, UEPP−D NPs showed no serious damage to the organs and histopathological comparison with the saline group revealed no obvious difference, confirming the safety of UEPP−D NPs. The serum levels of CK, ALT, AST, CRE, and BUN were found comparable with saline group (Figure 6B and Figure S2 in Supplementary Materials). Overall, these results indicate that UEPP−D NPs significantly decreased the DOX−induced hepatotoxicity and cardiotoxicity, further supporting their potential application for targeted breast cancer therapy.

## 4. Conclusions

To summarize, we established a promising exosomes−biomimetic nanoparticles for the efficient delivery of anticancer agents. Following intravenous injection, UEPP−D NPs exhibit prolonged blood circulation, effective anti−macrophage phagocytosis, enhanced tumor accumulation, and fast tumor microenvironment response release, leading to enhanced therapeutic effect of breast cancer with trivial side toxicity. Our study clearly demonstrates that exosomes from the patients’ urine have potential as cost−effective endogenous delivery vesicles to improve the anticancer efficacy.

## Figures and Tables

**Table 1:** Pharmacokinetic parameters of Free IR780, PP−IR780, and UEPP−IR780 NPs in MCF−7 bearing nude mice.

Groups	AUC_0→*t*_ (µg h/mL)	MRT_0→*t*_ (h)	*t*_1/2_ (h)	CL (mL/h/Kg)
Free IR780	37.48 ± 7.85	12.44 ± 2.16	8.72 ± 1.03	0.336 ± 0.085
PP−IR780 NPs	149.46 ± 32.53 ***	45.53 ± 7.27 ***	27.44 ± 3.18 ***	0.115 ± 0.022 ***
UEPP−IR780 NPs	207.54 ± 28.75 **	53.46 ± 6.35 **	31.25 ± 4.30 **	0.071 ± 0.021 **

*p* < 0.01 (**), *p* < 0.001 (***), Black asterisk for PP−IR780 versus free IR780, red asterisk for UEPP−IR780 versus PP−IR780.

## Data Availability

Not Applicable.

## Supplementary Materials

The following are available online at https://www.mdpi.com/article/10.3390/bioengineering9080363/s1, Figure S1. PDI values of the NPs (n = 3). Data are presented as mean ± SD; Figure S2. Serum levels of BUN and CRE treated with various groups (n = 5). Data are presented as mean ± SD.
