# Supplementary material for: Naturally Equipped Urinary Exosomes Coated Poly (2−ethyl−2−oxazoline)−Poly (D, L−lactide) Nanocarriers for the Pre−Clinical Translation of Breast Cancer
Source: Bioengineering (Basel). 2022 Aug 3;9(8):363. doi: 10.3390/bioengineering9080363 (PMC9404723; doi:10.3390/bioengineering9080363)
Supplement: Supplementary file 1 [file bioengineering-09-00363-s001.zip › bioengineering-1783248-supplementary.pdf]

bioengineering

MDPI

Supplementary Materials

Naturally Equipped Urinary Exosomes Coated Poly (2−ethyl−2−oxazoline)−Poly (D, L−lactide) Nanocarriers for the Pre−Clinical Translation of Breast Cancer

Jiang Ni [1], Yuanyuan Mi [1], Bei Wang [1], Yuting Zhu [1], Yang Ding [2], Yongjuan Ding [1,]* and Xia Li [1,]*

[1] Department of Pharmacy, Affiliated Hospital of Jiangnan University, Wuxi 214000, China; jiangni16401@163.com (J.N.); miniao1984@163.com (Y.M.); xuewuhenwang@126.com (B.W.); tingting9189@126.com (Y.Z.)

[2] College of Pharmacy, Pharmaceutical Series, China Pharmaceutical University, Nanjing 210000, China; dydszyzf@163.com

* Correspondence: jdfy1876@163.com (X.L.); dd8125767@163.com (Y.D.)

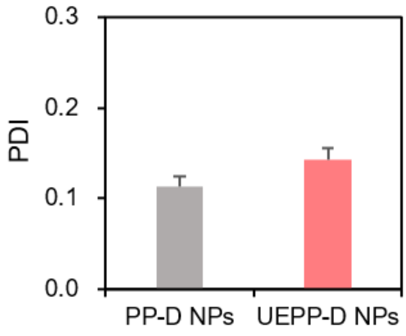

Figure S1. PDI values of the NPs (n = 3). Data are presented as mean ± SD.

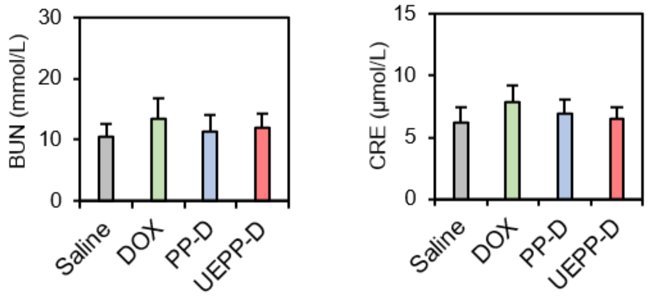

Figure S2. Serum levels of BUN and CRE treated with various groups (n = 5). Data are presented as mean ± SD.
